# Profile and risk factors in farmer injuries: a review based on Haddon matrix and 5 E’s risk reduction strategy

**DOI:** 10.3389/fpubh.2024.1322884

**Published:** 2024-06-06

**Authors:** Xuejie Qi, Xue Yao, Xianzhu Cong, Shuang Li, Mei Han, Zikun Tao, Xi Yang, Xiao Qi, Fuyan Shi, Suzhen Wang

**Affiliations:** ^1^Key Laboratory of Medicine and Health of Shandong Province, Department of Health Statistics, School of Public Health, Shandong Second Medical University, Weifang, China; ^2^Department of Interventional Vascular Surgery, China Rongtong Medical and Health Group Zibo 148 Hospital, Zibo, China

**Keywords:** injury, farmers, agriculture, risk factors, Haddon matrix, 5’Es risk reduction strategies

## Abstract

Farmers are considered a high-risk group for intentional and unintentional injuries. This review identified significant risk factors for agricultural injuries in farmers and explored injury prevention countermeasures based on the literature. Therefore, CiteSpace software was used to analyze the relevant literature in this field. Additionally, we identified both key risk factors and countermeasures using the Haddon matrix and the 5 E’s risk reduction strategies conceptual framework, respectively. The risk factors were identified from four categories (host, agent, physical environment, and social environment) corresponding to three phases (pre-event, event, and post-event). Interventions of 5 E’s risk reduction strategies including education, engineering, enforcement, economic, and emergency response have been proven effective in preventing injuries or reducing their severity. Our findings provide a comprehensive foundation and research direction for the study and prevention of injuries among farmers.

## 1 Introduction

There is increasing evidence suggesting that agriculture is one of the high-risk industries for occupational-related injuries (1). Injury is defined as the transfer of energy exceeding a certain threshold, resulting in harm to a person (2). Causes of injuries mainly include unintentional injury and intentional injury. The most common unintentional injuries among farmers are farm vehicle collisions, poisoning of biological and chemical substances, falls or slips, drowning, and burns (3). In particular, most accidents including farm vehicle collision are related to the use of machinery such as tractors. Therefore, researchers devoted to develop innovative protective structure and partial assistance system for equipping the protective structure of a track-laying tractor for the enhancement of work equipment safety (4, 5). In addition, a long-term pesticide exposure increased the risk of pesticide poisoning (6). Thus, it is important that occupational risk assessment of agricultural activities is related to the use of pesticides (7). Intentional injuries mainly involve violence against oneself or others such as suicide and homicide (8). Moreover, agriculture exhibits a higher rate of non-fatal injuries, leading to more lost health and workdays, decreased performance inefficiency, and an economic burden on farmers (9). The prevalence of injuries may be higher than realized due to minor injuries such as superficial cuts and bruises always being ignored (10). Additionally, the stigma associated with suicide in rural areas may lead to underreporting (11). Previous studies on farmers’ injuries have identified numerous risk factors defined as individual characteristics or exposures that increase the likelihood of developing injuries (12). Thus, engaging in agricultural work is directly related to health and occupational safety due to exposure to various risk factors. These risk factors include individual characteristics and work circumstance factors (12, 13). Actually, injuries are now regarded as largely predictable and preventable through effective safety interventions (14). Therefore, understanding the underlying mechanisms will contribute to the formulation and implementation of injury policies and interventions.

Many approaches have been proposed to investigate the mechanisms underlying injury reduction. In recent years, both the Haddon matrix and 5 E’s risk reduction strategies have gained increasing significance. The Haddon matrix was developed by William Haddon as a conceptual framework and has extensive applications in injury prevention and control (15–17). Integrating the epidemiologic triangle of host, agent, and environmental factors with three phases (pre-event, event, and post-event) facilitates identifying potential interventions that can be implemented (18). This model has been employed to conceptualize etiologic factors for injury and identify potential preventive strategies. Furthermore, the 5 E’s Risk Reduction strategy (including education, engineering, enforcement, economic incentives, and emergency response) was proposed by Sawyer et al. in order to prevent or mitigate the loss of life, property, and resources associated with life safety, fire, and other disasters within a community (19). This strategy can be also utilized to explore solutions for response to various injuries. Recently, Khan et al. proposed and implemented a combined model that integrates the Haddon matrix with the 5 E’s risk reduction strategies to elucidate the risk factors contributing to increased susceptibility and severity of COVID-19 infection while proposing prevention strategies (20). However, no studies have explored the measures for preventing, controlling, and mitigating farmers’ injuries using the Haddon matrix and 5 E’s risk reduction strategies model. The injury risk for farmers can vary depending on several factors, including individuals’ characteristics, the use of farming machinery and tools, the type of work being performed, and the work environment. Therefore, our current study summarized significant risk factors from different hierarchy and stage. It is contributed to put forward potential injury prevention approaches according to the hierarchy of control.

In this review, we conducted a bibliometric analysis to identify and summarize the research frontiers and hotspots related to risk factors of farmer injury in high-income countries from 2003 to 2023. Additionally, we employed the Haddon matrix conceptual framework to identify key risk factors related to injuries occurred during agricultural work. Finally, we explored suitable responses and control measures based on these identified risk factors by integrating the 5 E’s risk reduction strategies. It is anticipated that the findings of this study will provide valuable research insights and guidance for interventions targeting farmer injury in high-income countries.

## 2 Methods

### 2.1 Search strategy

**Figure 1:**
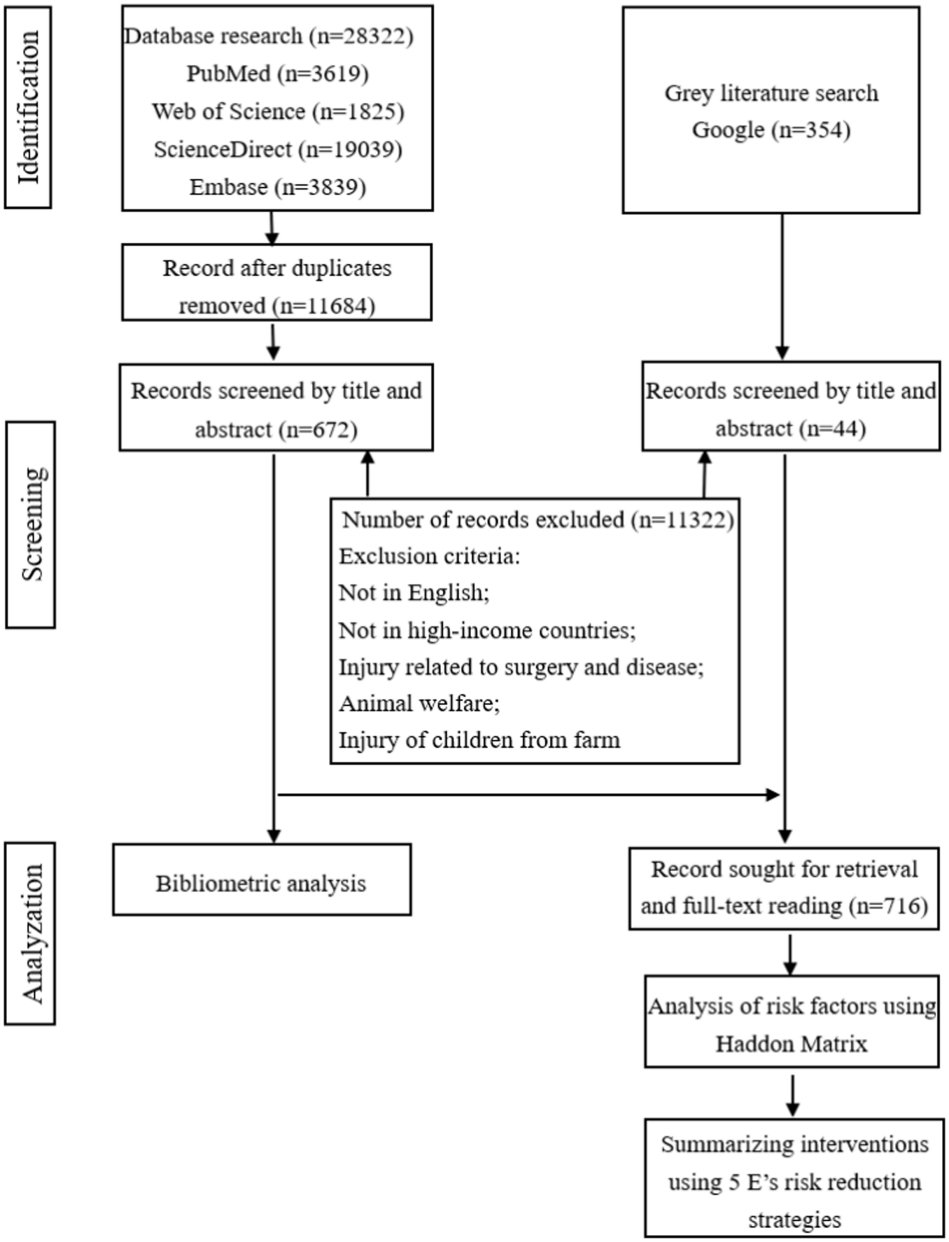
The research process view.

The literature types encompass peer-reviewed articles, reviews, conference proceeding papers, and gray literature. We conducted our search using online databases including PubMed, Web of Science, ScienceDirect, and Embase. Additionally, we performed a Google search using keywords (farmer, risk factor, and injury) to identify relevant gray literature which was further reviewed after the initial search. We determined the research period for this study to be from 1 January 2003 to 31 December 2023. The search formula used in this study is shown in Table 1. Studies included in this review were limited to those conducted in high-income countries as defined by the World Bank.^1^ Subsequently, duplicates were eliminated using EndNote X9.3 software. Following this step, the literature was screened based on an initial reading of titles and abstracts. Ultimately, 716 texts were included in the review; among them, 672 texts were utilized for bibliometric analysis. Flow diagram for article selection and analysis was shown in Figure 1. The review process was conducted independently by two reviewers following the same method to minimize bias.

**Table 1:** Retrieval formula of keywords.

Search	Query
#1	(farmer) OR (agriculture)
#2	(risk) OR (risk factor)
#3	(injury) OR (harm)) OR (accident)) OR (hurt)) OR (wound)
#4	((#1) AND #2) AND #3

### 2.2 Inclusion and exclusion criteria

The inclusion criteria included the following: (1) farmers aged between 18 and 85 years; (2) publications from high-income countries; (3) publications in the English language; and (4) studies related to the risk factors associated with injuries among farmers engaged in agricultural work; (5) research period from 2003 to 2023.

Our exclusion criteria included the following: (1) injuries related to surgery and disease; (2) studies related to animal welfare; and (3) occupations not involving agriculture or solely involving family members of farmers.

### 2.3 Bibliometric analysis methods

Following the screening, we conducted an analysis of co-occurrence of keywords and timeline clusters using CiteSpace 6.2.R4 software (21). Co-occurrence network analysis of keywords refers to two or more keywords appearing together in a document. In addition, analysis of keywords can indirectly reveal various critical research topics and characteristics of a research field. In terms of parameter setting, the timespan was set to “from January 2003 to December 2023, 1 year per slice.” The node type was set as “keyword.” Link strength was selected as “cosine (cosine function).” Selection criterion was selected as “TOP N,” selected top 15 levels of most cited or occurred items from each slice. To make the structural network clearer, keywords with OR ≥ 7 and *p* ≤ 0.01 were selected. We have used the tailoring strategy of Pruning Sliced networks in this article and required keywords to have a minimum duration of 2 years. CiteSpace can generate clusters of keywords to predict the research frontiers and emerging trends in this field. The log-likelihood ratio (LLR) method was used for keyword clustering. The cited frequency and centrality of the nodes were calculated. The centrality indicates the significance of nodes in a network, and the higher the centrality, the more significant they are. In the map, nodes with a centrality ≥0.1 were marked with purple circle. The nodes represent the analyzed keywords, with their size proportional to their frequency. The color of the nodes corresponded to the time of their initial co-occurrence or co-citation, transitioning from cooler tones to warmer tones as time progresses from early to recent. The network density is an indicator that measures the strength of a network. When the number of connections between all nodes exceeds the actual distances between them, it indicates that the network is tightly connected and robust. In general, a network density ranging from 0.05 to 0.1 can be considered within a reasonable range. S (silhouette) indicates the average contour value, which serves as the basis for judging the effectiveness of map drawing. Clustering is generally considered reasonable when S > 0.5, and it has high reliability when S > 0.7. Q (modularity) indicated module value, which serves as the basis for judging the effectiveness of graph drawing. The clustering result is significant when Q > 0.3. Additionally, we used Microsoft Excel 2016 for data management to examine yearly publication trends and country-specific distribution.

## 3 Results

### 3.1 Publication dynamics

**Figure 2:**
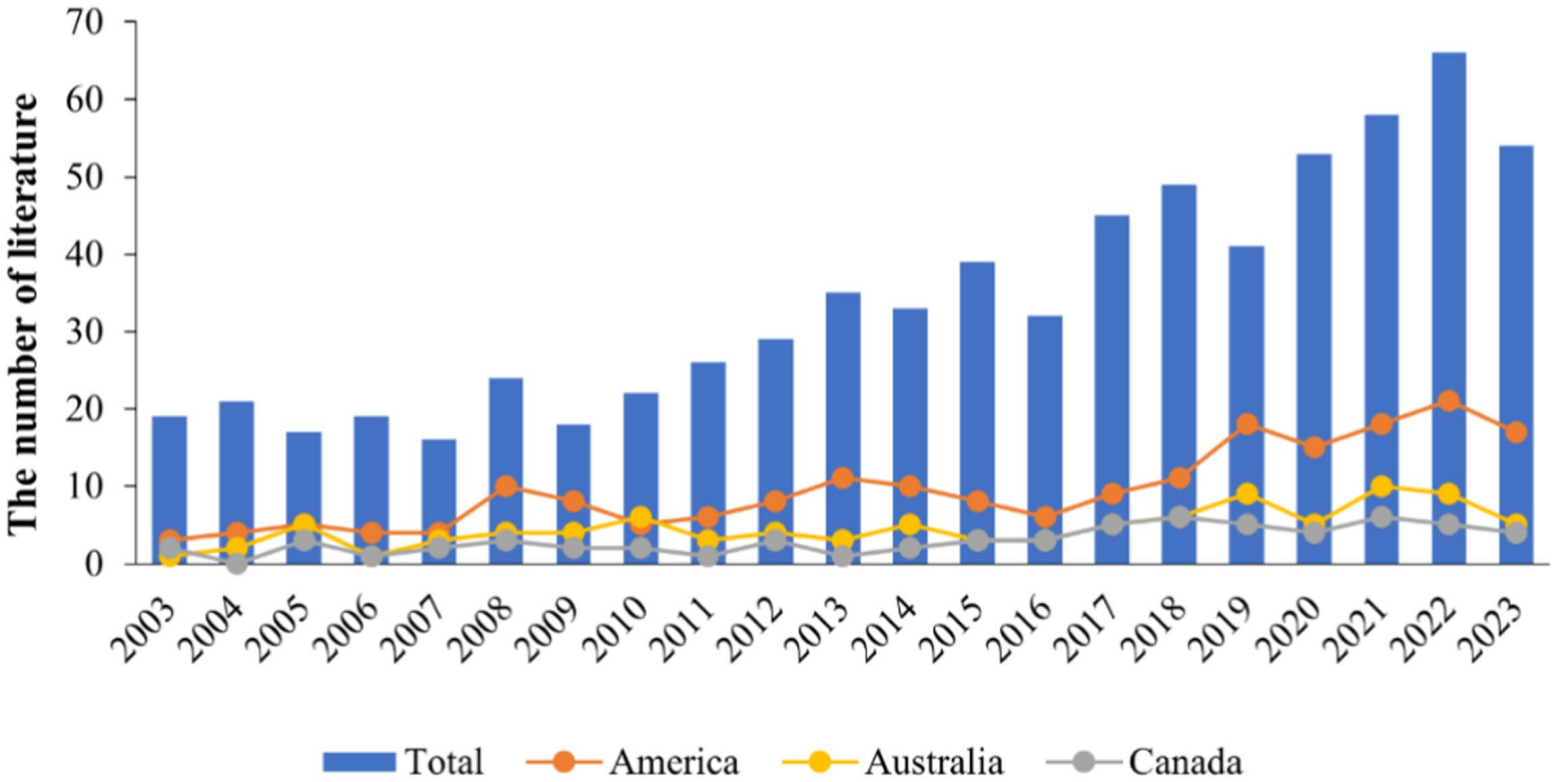
Publication outputs and time trend.

The article included 716 literature studies. The findings demonstrate that the United States, Australia, and Canada rank as the top three countries in terms of publications related to risk factors associated with farmer injuries. The temporal trend in publication numbers from 2003 to 2023 is shown in Figure 2. The top 20 countries based on publication count are shown in Supplementary Table S1.

### 3.2 Co-occurrence analysis of keywords

**Figure 3:**
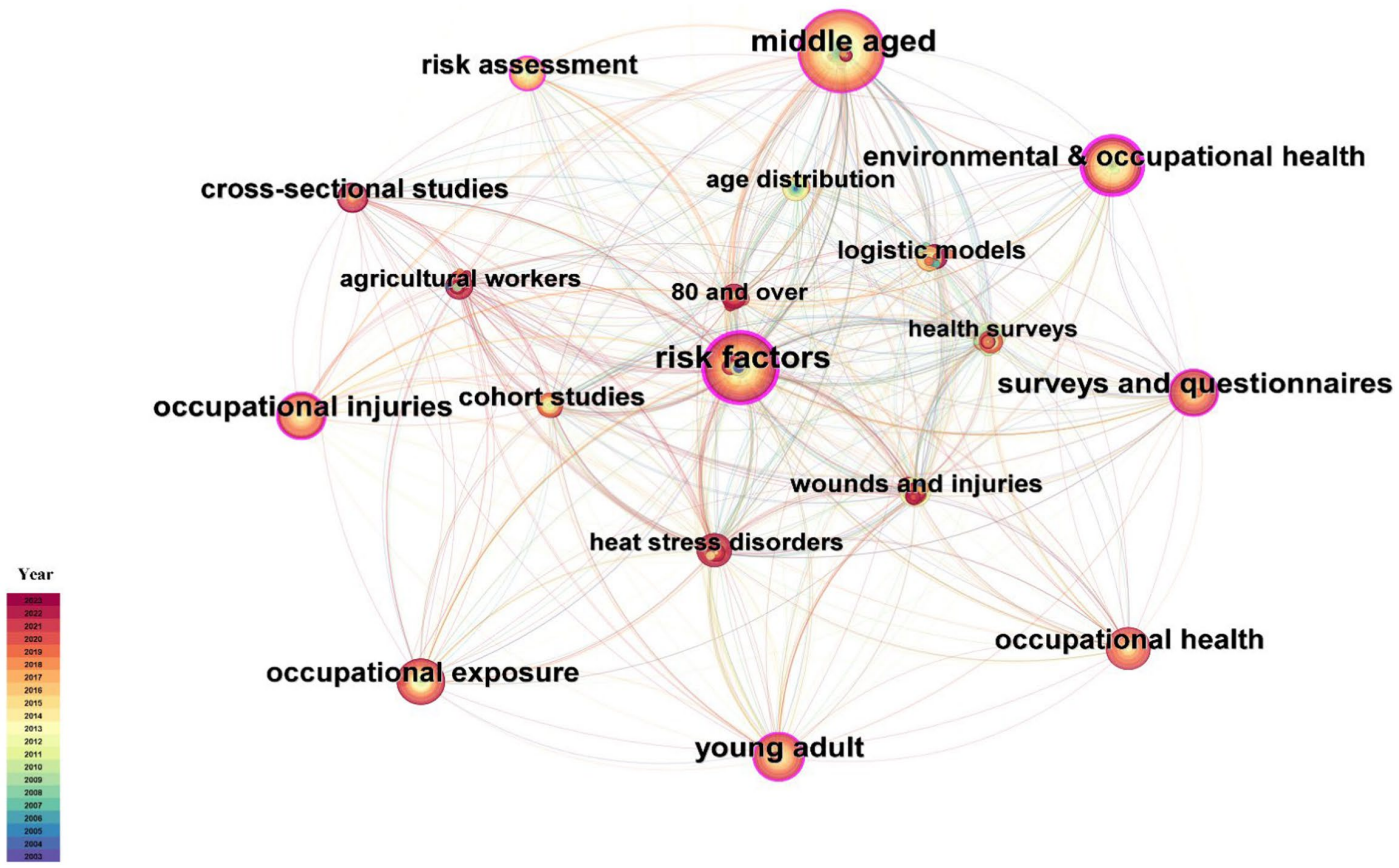
Keyword co-occurrence view of risk factors in farmer injuries from 2003 to 2023. The nodes represent the keywords, and the more the frequency, the larger the nodes. The color and thickness in the inner circle of the node indicated the occurrence or cited frequency of different time periods. The nodes with centrality ≥0.1 were marked with purple circle.

**Figure 4:**
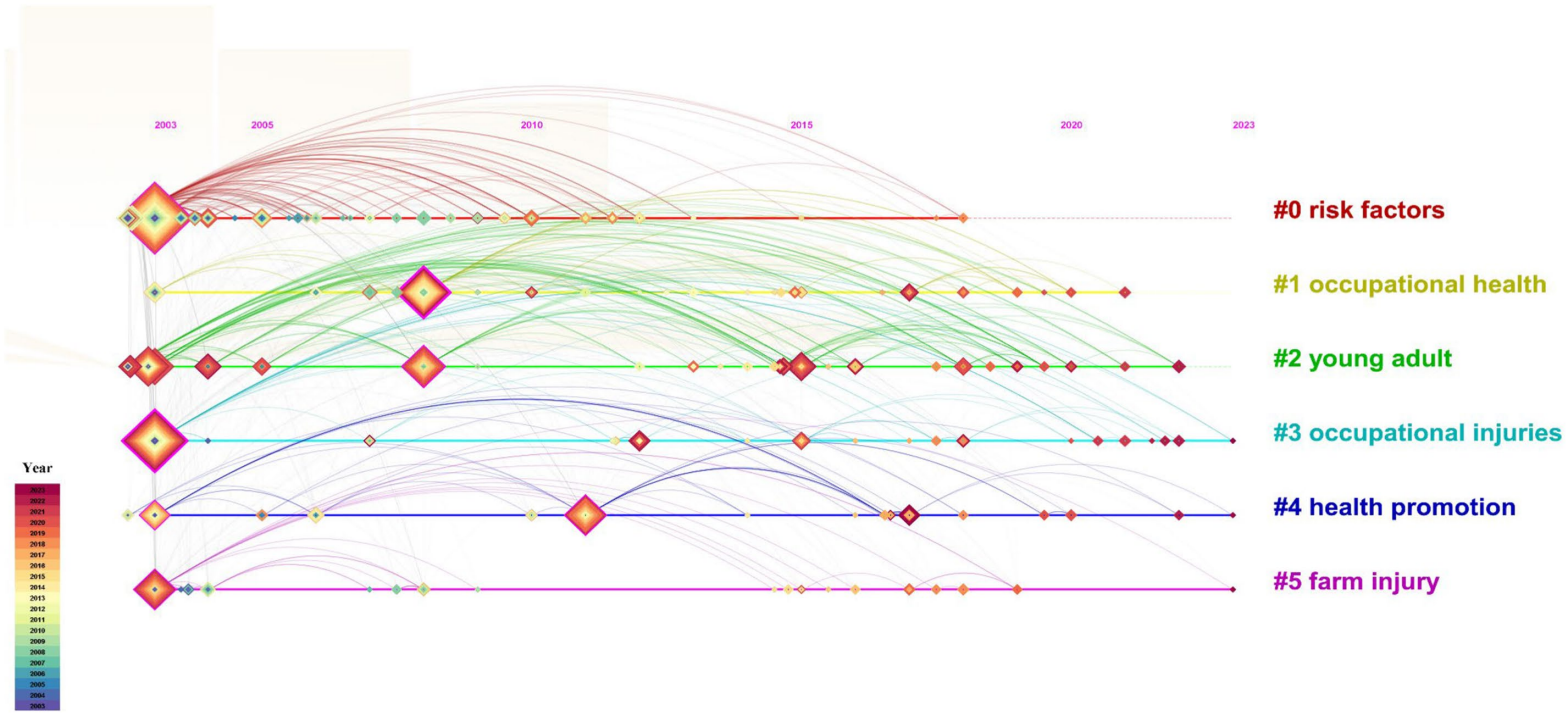
The timeline visualization of the keywords cluster from 2003 to 2023. The nodes represent the keywords, and the more the frequency, the larger the nodes. The color and thickness in the inner square of the node indicated the occurrence or cited frequency of different time periods. The nodes with centrality ≥0.1 were marked with purple circle. Cluster label with different colors represent different clustering groups.

To comprehend the prevailing topics, we employed CiteSpace to conduct a co-occurrence analysis of high-frequency keywords, as shown in Figure 3. Subsequently, 212 nodes and 1,190 links were obtained with a network density of 0.0532. The top 20 keywords with high frequency are shown in Table 2. Simultaneously, the keywords were clustered based on timeline distribution to effectively unveil the changing trends over time using the LLR algorithm (Figure 4). The modularity Q was calculated as 0.3479, which is >0.3, indicating a noteworthy cluster structure significance. Furthermore, with a mean silhouette value exceeding >0.5 at 0.7014, it demonstrated that our clustering approach was reasonably sound and reliable. Finally, we obtained six clusters (Table 3).

**Table 2:** Top 20 keywords with high frequency in keyword co-occurrence analysis (2003–2023).

Rank	Keyword	Frequency	Centrality	Year
1	Middle aged	179	0.19	2003
2	Risk factors	153	0.38	2003
3	Environmental & occupational health	86	0.24	2008
4	Surveys and questionnaires	81	0.16	2003
5	Occupational health	75	0.10	2003
6	Young adult	69	0.11	2008
7	Occupational exposure	60	0.07	2003
8	Occupational injuries	58	0.15	2011
9	Cross-sectional studies	39	0.07	2004
10	Risk assessment	38	0.13	2003
11	Wounds and injuries	32	0.02	2003
12	Heat stress disorders	31	0.01	2015
13	Cohort studies	24	0.04	2003
14	Logistic models	21	0.03	2003
15	80 and over	18	0.01	2003
16	Health knowledge	18	0.02	2005
17	Health surveys	16	0.01	2003
18	Agricultural workers	15	0.01	2003
19	Age factors	14	0.01	2003
20	Farm safety	12	0.02	2003

Year: The earliest time that occurrence of keywords.

**Table 3:** Information of keyword clusters.

Cluster ID	Size	Sihouette	Top five terms (log likelihood ratio, *p* value)
#0	43	0.721	risk factors (36.68, *p* < 0.001); age distribution (31.83, *p* < 0.001); sex distribution (23.12, *p* < 0.001); logistic models (17.33, *p* < 0.001); retrospective studies (15.76, *p* < 0.001)
#1	33	0.717	occupational health (45.32, *p* < 0.001); health care sciences (33.07, *p* < 0.001); farm injuries (32.35, *p* < 0.001); agricultural task (24.15, *p* < 0.001); environmental sciences (20.93, *p* < 0.001)
#2	33	0.783	young adult (48.61, *p* < 0.001); cross-sectional studies (37.31, *p* < 0.001); agricultural workers (31.24, *p* < 0.001); hot temperature (25.61, *p* < 0.001); occupational exposure (25.55, *p* < 0.001)
#3	32	0.618	occupational injuries (22.15, *p* < 0.001); work-related injuries (19.54, *p* < 0.001); sustainable agriculture (11.06, *p* < 0.001); musculoskeletal disorders (10.06, *p* < 0.01); retrospective studies (7.37, *p* < 0.01)
#4	24	0.826	health promotion (29.09, *p* < 0.001); cohort studies (17.6, *p* < 0.001); injuries (17.53, *p* < 0.001); risk assessment (17.53, *p* < 0.001); occupational medicine (17.35, *p* < 0.001)
#5	24	0.520	farm injury (20.37, *p* < 0.001); disability evaluation (16.47, *p* < 0.001); health disparities (12.91, *p* < 0.001); protective devices (11.69, *p* < 0.001); attitude to health (10.08, *p* < 0.01)

Size: the total number of terms in each cluster.

### 3.3 Application of Haddon matrix to risk factors of injuries in farmers

The key risk factors identified in the literature were assessed using the Haddon matrix, as presented in Table 4. The Haddon matrix consists of four columns (host, agent, physical environment, and social environment) and three rows (pre-event, event, and post-event). Specifically, the host refers to an individual affected by injury; the agent is defined as the energy transferred to the host by an inanimate or animate vector; the physical environment encompasses various elements (e.g., road and climate) in the surrounding area; and the social environment refers to the sociopolitical milieu including cultural or economic factors.

**Table 4:** Haddon matrix applied to risk factors of farmer injuries.

Event phases	Influencing factors of farmer injurie			
	Host	Agent	Environment	
			Physical	Social
Pre-event	• Age (22–25)	• Equipment/Tool (26)	• Design of workplace (6)	• Economic level (27, 28)
	• Gender (29–33)	• Agricultural vehicles (34–36)	• Climate conditions (e.g., rainfall/ lightning/heat/ cold/ radiation) (37, 38)	• Rules and regulations of work organization (e.g., work task, work time, safe standard) (9, 38, 39)
	• Literacy level (40, 41)	• Chemical, physical and biological substances (42, 43)		•Farming native culture (44–47)
	• BMI (48, 49)	• Sprayer (50)		• The policy and legislation (51–55)
	• Regularly medicine (56)	•Animal handling (57–60)		
	• Alcohol abuse (61)	•Firearm (37, 51, 62, 63)		
	• Fatigue (64)			
	• Work-related pressures (65)			
Event	• Non-technical skills (e.g., situation, decision-making awareness, teamwork, etc.) (66–69)	• Personal protective equipment (70, 71)	• Escape routes, refuges, shade or rest space (72)	• The values of agrarianism (independent work, group conformity, peer support) (73, 74)
	• Risk perception (75)	• Protective equipment of vehicle (76, 77)	• Outdoor environment (38)	
	• Adaptive decision (78)	• Mitigation devices (79)	• Supervision of families and co-workers (80, 81)	
	• Injury experience (29, 78, 82)	•safety signs or warning signs (83)		
Post-event	• Physical vulnerability (84)	• Medical resource (e.g., medical service, first-aid supplies, ambulance) (85)	• Isolated geographic position (86, 87)	• Social trauma systems (88, 89)
	• The resilience (90, 91)	• Compensation insurance (71)	• Emergency medical service (92–96)	• The government income protection policy (97, 98)
		• Communication equipment (99)	• Clear road that allows access for emergency vehicles (35)	• Medical economic burden (100)
				• Agrarian community ideology (101, 102)

#### 3.3.1 Pre-event

##### 3.3.1.1 Host

At the host level, age, gender, literacy level, and body mass index (BMI) have been identified as significant risk factors for farmer injuries (22). First, evidence suggests that older farmers (aged 65–85 years) have a lower incidence of farm-related injuries; however, they experience higher fatality rates, severity, and injury cost compared with younger farmers (23). Conversely, young adult farmers (aged 18–44 years) and middle-age farmers (aged 45–64 years) are generally more susceptible to non-fatal injuries due to their inclination toward risk-taking tendencies and greater propensity for sensation-seeking activities (24). They are more likely to engage in unsafe operating practices and expose themselves to inherently hazardous situations (25). Second, men generally exhibit a higher rate of injury occurrence compared with women (29, 30). This can be attributed to their involvement in heavy machine work and longer working hours on farms compared with women (31). The characteristic of injuries also differs between male and female workers due to variations in job tasks and risk perceptions. Male farmers have a higher risk of transportation-related injuries, fall from a height, rollovers, and animal handling due to their risk-taking behaviors and lifestyles (29). However, studies showed that most of the farmers who died by suicide were men while the farmers who attempted suicide were mostly women (32, 33). Third, farmers with lower educational attainment face a high risk of agricultural work-related accidents (40). Lower education level is consistently found among women, older, and low-income farmers who tend to lack awareness regarding the importance of agricultural safety (41). Fourth, the study demonstrated that an increased BMI is associated with a higher risk of injuries (48). A high BMI imposes a greater musculoskeletal burden and restricts farmers’ mobility, potentially leading to fall-related injuries (49). Furthermore, the use of psychotropic medication and alcohol can impair workers’ ability to respond promptly to stimuli and risk awareness (56). The research also found a positive correlation between alcohol abuse, certain psychotropic medication usage, and unintentional injuries such as traffic collisions and falls (56, 61).

Finally, both elevated levels of pressure and fatigue have been identified as risk factors for farm vehicle collision and suicide in the agricultural sector (64, 65). The persistent heavy physical workloads, overexertion, financial challenges, and climate variability may contribute to fatigue and mental health disorders. Among farmers, inadequate management or coping strategies for these pressures can significantly increase the risk of suicide. Additionally, sleep disorders have been reported as an important potential risk factor for agricultural injuries that are subsequently sustained on farms (84).

##### 3.3.1.2 Agent

Agricultural vehicles (e.g., tractors, harvesters, quad bikes, and all-terrain vehicles) and equipment play a crucial role in causing injuries among farmers. Research has demonstrated that vehicle crashes often result in severe head and facial trauma (34). Agricultural vehicle crashes, loss of control, and lateral rollovers, as well as workers falling or slipping off the tractor are major causes of injury and fatality for farmers (35). Furthermore, unstable design and limited perceptibility of farm vehicles increase the likelihood of rollover and collision (36). Agriculture work also involves the use of various types of hazardous equipment. Improper usage of farming equipment or tools significantly increases the risks of injuries (e.g., accidental punctures, cuts, fractures, or lacerated wounds) and fatalities (26).

Farmers frequently contact with a variety of chemical, physical, and biological substances such as pesticides, agricultural dust, and microorganism. These substances can unintentionally be exposed through inhalation via the respiratory tract, ingestion through oral routes, or dermal contact during pesticide application processes, resulting in poisoning (42, 43). The use of sprayers has been reported as an important contributing factor in increasing pesticide poisoning cases since droplets sprayed by sprayers can be inhaled or come into dermal contact (50). Furthermore, organic dusts, microorganisms, and agricultural products such as wheat and seaweed are often stored in home, which further increases the risk of poisoning.

The handling of animals, especially large ones, can result in severe injuries and fatalities for livestock farmers (57). Repeated contacts with animals (such as milking, feeding, hoof trimming, and moving) increase the risks of being pushed, kicked, crushed, bitten, or headbutted by workers due to unpredictable behavior and heightened levels of animal stress (58). Furthermore, injuries related to domestic animals (e.g., snakebites or dog bites) and their associated fatalities remain prevalent within the agricultural workforce (59, 60).

Firearms play a significant role in causing injuries among farmers, with higher rates of firearm-related injuries and mortality rates observed in certain countries such as the United States (62) and several European countries (51, 63). The accessibility of firearms is particularly pronounced in rural areas, where farmers are more likely to possess them. Furthermore, a study has reported that rural regions exhibit elevated rates of unintentional and intentional firearm-related death (e.g., homicide and suicide) among farmers (37).

##### 3.3.1.3 Physical environment

On the one hand, the inadequate physical design of the workplace can exacerbate underlying conditions related to accidents (10). For instance, uneven slopes and narrow, slippery pathways increase the likelihood of farmers slipping, tripping, and falling in their work environment. These poor roadway conditions, combined with limited visibility and obstacles, can also impede safe tractor operations leading to crashes or overturn injuries. On the other hand, outdoor workers tend to endure prolonged exposure to severe weather conditions, including rainfall, lightning strikes, heat, and sun exposure. This adverse climate environment heightens the risk of fall and lightning strike incidents (38). Moreover, research conducted in Australia has revealed that extreme climate change along with crop damage and loss contributes to an increased incidence of suicide among farmers (103).

##### 3.3.1.4 Social environment

Farming is predominantly a self-employed occupation with a low degree of formal organization. The lack of regular work arrangements, such as prolonged engagement in physically demanding tasks, can increase the risk of injury for farmers (39). For example, a study showed that the odds of injury gradually increased with longer exposure to farm-related work (13). Hagen et al. have documented that enduring challenging working conditions and an intense workload over time may lead to mental health burdens such as anxiety and stress, which are widely acknowledged as significant contributing factors to suicide cases (44).

Farming safety culture primarily involves the establishment of safety regulations, family parenting practices, and acceptance of firearms (45). Implementing farm-related safety rules and controls, such as adhering to safety protocols and utilizing appropriate protective gear, is crucial in fostering a comprehensive safety climate at both individual and group levels to effectively prevent injuries (46). It has been observed that parents who exhibit lax-inconsistent disciplining tend to have a positive association with various unsafe farm behaviors displayed by their children (25). Moreover, farmers’ easy access to firearms for hunting purposes within the cultural context of accepting firearms contributes to a higher percentage of firearm-related injuries among this occupational group (27, 47).

In addition, the economic level is also an important factor that affects farmers’ risk of injury. On the one hand, farmers in high-economic countries have greater access to mechanization and advanced technology. However, it can increase the likelihood of traffic injuries or mechanical force injuries such as cuts, crushes, and crashes (28). On the other hand, higher income levels for a farming family may lead to an increase in work tasks and exposure situations, thereby increasing the possibility of injury (104).

#### 3.3.2 Event

##### 3.3.2.1 Host

Non-technical skills (NTS), such as situation awareness, decision-making, teamwork, and communication, play a crucial role in mitigating the severity of injuries among farmers during events (66, 67). The ability to maintain situation awareness regarding operating systems, agricultural vehicles, and environmental conditions empowers farmers to make timely and informed decisions that can effectively reduce the extent of injury (68). Furthermore, proficient leadership skills and effective communication among farmers when accidents occur have the potential to significantly minimize the consequences of injuries (66). Additionally, ensuring seamless communication facilitates effortless exchange of information on employees’ whereabouts and enables timely updates on injury statuses for fellow team members (69).

Risk perception is considered as an individual’s subjective evaluation of the potential consequence of an accident when an event occurs (75). Farmers who possess the ability to perceive risks can better understand the characteristics and extent of encountered hazards, enabling them to respond effectively in their future behavior. In general, farmers tend to manage injury events and associated risks through adaptive decision-making, drawing from previous injury experience (78). Several researchers have observed that farmers with a history of serious injuries are less likely to experience subsequent injuries among workers (29). This may be attributed to the development of improved farm safety features and systems following past injury incidents. Moreover, farmers who have experienced serious injuries are more likely to exhibit increased safety behaviors and response capacity (82).

##### 3.3.2.2 Agent

Personal protective equipment (PPE) serves as a highly effective control measure during events to mitigate the risk of severe or fatal injuries sustained by farmers (70). For example, drivers who buckle their seatbelt can effectively evade ejection from vehicles or collisions with the rollover bars in the event of a crash or overturn (71). Commonly used PPE includes helmets, goggles, gloves, and protective shoes. Furthermore, equipping agricultural vehicles with specific protective equipment could significantly reduce the consequences for the driver (76). Rollover protective structures (ROPS) and seatbelts are considered the most efficacious means of preventing operator fatalities and injuries. As illustrated, Bruno et al. designed an innovative ROPS model mounted on the tractors to enhance tractor stability and minimize driver injury risks during rollover incidents (77). In addition, safety signs, warning labels, and mitigation devices are the preferred means to alert operators about residual risks and facilitate the development of safe behaviors. This includes emphasizing attention to slope steepness, implementing an effective light marking system, and employing machinery-related usage risk signs (79, 83).

##### 3.3.2.3 Physical environment

People may miss valuable survival time if they are unable to promptly locate viable escaping routes or refuges. Many serious injuries can be mitigated with the implementation of improvised escape routes, safe havens, shade areas, and rest places within agricultural workplaces that can significantly alleviate the severity of injuries (72). Outdoor workers are expected to reduce the risk of lightning strikes or sunstroke if they are sheltered in these safe areas. Moreover, outdoor agricultural workers are particularly vulnerable to ambient temperature, especially when injured. Farmers have limited opportunities for self-protection while exposed to hot, humid, or frigid environments (38).

##### 3.3.2.4 Social environment

The traditional custom of agrarian values and native culture, which encompasses independent work, group conformity, peer support, and family farm culture, can affect individual knowledge or understanding of risk sources among farmers and shape their attitudes toward risk (73). First, farmers often engage in solitary without direct supervision from safety supervisors or team workers (74). Consequently, when faced with injury events, these independent farmers may be more likely to go unnoticed and be unable to seek help. A study suggested a significant association between supervisors’ tolerance for risk-taking and unsafety behaviors exhibited by young workers (24). Second, group conformity implies that the unsafe behavior of peers or family members may impact most workers. Peer support has been proven as an effective social support mechanism in mitigating suicide risks among farmers (80). Moreover, the family farm model represents a prevalent farming pattern in farming that is associated with the incidence rate of unintentional injuries (81, 105).

#### 3.3.3 Post-event

##### 3.3.3.1 Host

Physical vulnerability, including an individual’s perception and response to disability, underpins the process of adaptation following injury. Resilience is also a crucial factor in facilitating recovery from injury. Farmers often require more time to recover and heal after being injured. Factors that contribute to resilience in the face of injury include positive self-appraisal, positive emotions, and adaptability for many farmers to continue working without fully recovering, which can impede their overall rehabilitation (90, 91).

##### 3.3.3.2 Agent

A comprehensive medical resource, including ambulance service, medical personnel, equipment, and facilities, has facilitated farmers’ access to trauma care following accidents. However, research indicates that residents in remote rural areas may face challenges in timely ambulance transportation and subsequent access to effective healthcare (85). Additionally, the importance of communication equipment in facilitating timely access to trauma care for the injured has been emphasized by several researchers. Moreover, farmers often carry communication devices to maintain contact and monitor their location and personal status when working alone or in remote areas (99). Although workers’ compensation insurance can provide financial assistance during the recovery phase, many self-employed farmers remain uninsured (82), creating a potentially dangerous situation where farmers may continue to work after sustaining an injury.

##### 3.3.3.3 Physical environment

The geographic position of farms often results in isolation and distance from hospitals, presenting significant obstacles for farmers seeking timely trauma care after sustaining injures (86). First, they may have to travel considerable distances to reach a hospital. Second, emergency medical systems may not be able to promptly transport injured farmers due to their remote location or inconvenient transportation conditions, leading to delays in emergency care. Consequently, agricultural workers are at a greater risk of long-term disability or death following an injury compared with other occupational workers (87). In this context, the establishment of emergency shops near farms could provide crucial first aid and resources for injured farmers in rural communities while facilitating referrals to the hospital.

##### 3.3.3.4 Social environment

The evaluation of injury severity and subsequent determination of appropriate care levels are standardized in trauma systems. These systems effectively provide trauma care for a diverse population with multiple injuries, thereby contributing to improved outcomes following an injury (88). A systematic review has demonstrated that the implementation of trauma care is associated with an estimated 15% reduction in mortality among injured patients (89).

The government’s income protection policy can enhance support for farm management during the recovery process. Otherwise, farmers may be compelled to work under less-than-ideal physical conditions due to concerns about economic burden (97). The increasing financial burden on farmers may potentially impact their willingness to seek treatment for injuries. For example, to reduce economic losses, many low-income farmers may continue working despite tolerable injuries (98), thereby lacking sufficient disposable resources for prompt medical care.

Under the ideology of agrarian community, farmers would persist in working during the recovery phase with the assistance of their family, friends, and the local farming community (101). To navigate through injury, parents rely on families as protectors to facilitate injury recovery and seek solace for coping with losses (100). However, a survey revealed that most farmers rarely consider the future impact of injuries and are reluctant to seek mental healthcare without familial support (102).

### 3.4 Intervention strategies using the 5 E’s risk reduction strategies combined with Haddon matrix

We further investigated the intervention strategies for injury prevention in farmers by employing 5 E’s risk reduction strategies, which include education, enforcement, engineering, economic incentives, and emergency response combined with the Haddon matrix (Table 5).

**Table 5:** The intervention strategies of 5 E’s by combined strategies.

Intervention strategies	Timeline	Prevention and control strategies		
		Host	Agent	Physical and social environment
Education	Pre-event	• Increasing the perception and willingness of receiving safety production	• Education about operating safely agricultural vehicles and equipment	• Government incentives for education program
		• Propagandization of reduce alcohol consumption	• Correct application of pesticide and sprayer use	• Village-visit education services for older farmers
			• Increasing the knowledge about biological control	•Agricultural council provide agriculture-related safety operating manuals
			• Safe storage of firearms and toxic substance	regularly survey potential farm safety hazard
			• Safety training on animal handling	
	Event	• The direction of strengthen response capacity and NTS	• Innovative agriculture technology use training	• Positive safety behaviors education for family and peer
			• Correct PPE use	
	Post-event	• Educated farmer to seek timely appropriate trauma care	• Rural medical staff emergency training	NA
		•Standardized first aid training		
Engineering	Pre-event	• Develop safety education intervention model (e.g., AR 3D simulator) for farmers	• Design of hand tools such as increasing handle length and covering soft cushion, etc.	• The improvement of farm road and traffic
			• Innovative agricultural device with elimination, substitution and engineering controls	
			• Development of newer machinery and vehicle (e.g., optimize the stability of vehicle)	
	Event	NA	• The improvement of individual and equipment’s protective device (e.g., ROPS, PTO on tractor)	• The establishment and application of escape place or refuges in workplace
	Post-event	NA	NA	NA
Enforcement	Pre-event	• Formulation of work rotation system for workers	• Legislation for firearms, pesticide and hazardous materials abuse and storage	• Conduction of routine and unannounced inspections for workplace
		•Enforcement of drunk driving restrictions		• Regular risk assessments for workplace
				• Workplace physical environment regular risk assessments and inspections
	Event	NA	• The enforcement and legislation about individual and agricultural vehicle PPE	• Regulation on construction requirements and machinery states
			• Increasing warning signs in higher risk place	
	Post-event	• Routine annual medical examinations of injured workers and necessary health services	NA	• Admissible management policy for injured farmers including formulation of work rotation system and appropriate break, etc.
		• Management policy about health services were provided for injured farmers		
Economic Incentives	Pre-event	• Subsidizing investments in occupational safety education and training for farmers	• The investments in technology, safety equipment, infrastructure and constructions	• Financial support for safe workplace construction by government
	Event	NA	• Free provision of necessary PPE for farmers	NA
	Post-event	• Medicine and physical examination subsidies for injured farmers	• Supplement and preparation for medical resources	• Subsidizing occupational safety private or commercial health insurance
				• Government subsidies for health care services and trauma system
Emergency Response	Pre-event	• Provision emergency cards for farmers	• The regular check and evaluation for vehicle and equipment	• The improvement of agricultural injury surveillance systems
		• Emergency preparation in daily farming work	• The accessibility of PPE in workplace	• Workplace safety monitoring and risk assessment
	Event	• Summarizing experiences and making preparation in daily farming work	• Preparation and practices of rural health workers and medical resources	NA
		• Access to effective risk communication	• The improvement of public health and medical service facilities	
	Post-event		• Improvement of emergency medical services	• Effective risk assessment and task management for farm condition

#### 3.4.1 Education

The higher incidence and mortality rate are associated with farmers who have a low educational background (106). Therefore, we synthesized various education strategies in conjunction with the Haddon matrix. To prevent injuries, it is imperative to implement safety education programs tailored for farmers and their families, particularly targeting high-risk groups. Numerous studies have demonstrated that agricultural health and safety education can enhance risk perception and promote preventive behaviors (41). For example, interventions such as reducing alcohol consumption, ensuring proper pesticide application, promoting biological control, and addressing drug abuse should be disseminated through farmer field schools, advertising campaigns, mass media platforms, or social networks in order to effectively reduce injury prevalence (107). Enhanced safe storage practices for firearms and toxic substances are crucial in reducing the risk of unintentional incidents. To ensure self-protection, it is important to store firearms unloaded, separate from ammunition, and securely locked using an appropriate device (108). Therefore, widespread education and intervention programs should be implemented to promote safe storage of dangerous goods. Additionally, farmers should receive regular training on identifying potential safety hazards within their work environment. Furthermore, comprehensive safety workshops or small group formats should be conducted to educate farmers on proper animal handling techniques such as maintaining gentle contact, keeping a safe distance, and avoiding sudden behaviors that may startle them, ultimately reducing the risk of injury (109). Moreover, farmers can improve their vigilance and alertness toward the risk of injuries through NTS training (110). It is essential to customize formal NTS training programs to raise awareness about risk controls, behavioral modifications, and emergency response strategies (111). Specially, situation awareness training should also be developed as a pivotal component of NTS interventions tailored to different agricultural tasks based on survey findings from the target population.

Once events occur, PPE serves as a crucial control measure for mitigating injuries. Educated farmers were more likely to adopt novel technology or protective interventions to facilitate their work compared with those with limited education (112). The primary barriers identified by farmers were discomfort, time wastage, and inconvenience associated with donning and doffing PPE. Therefore, researchers should consider these potential barriers and propose strategies to motivate farmers to actively and correctly use PPE. In addition, given the influence of family farming culture on safety education for younger farmers, it is crucial to emphasize positive parental or peer education as a way to cultivate safe work habits and enhance the perception of safeguard procedures among farmers (113).

Most importantly, it is imperative to enhance education on trauma care perception to encourage farmers to seek appropriate medical assistance. Specifically, agriculture-related guidance and operating manuals should be provided for each farmer by the agricultural council. For older farmers who have difficulties reading and comprehending safety guideline manuals, village-visit education services could be implemented (114). These proactive measures of education and training are expected to foster behavioral changes and cultivate a positive attitude toward farm safety, ultimately leading to the reduction and mitigation of accidents and injuries related to farming activities.

#### 3.4.2 Engineering

The integration of new technology or equipment can not only enhance the optimization of agricultural practices but also mitigate the risks associated with agriculture-related injuries. First, incorporating ergonomics interventions can effectively reduce the likelihood of strains and sprains, particularly among younger farmers (115). Recent research has shed light on emerging human-robot synergy systems aimed at minimizing work-related injuries (116). For example, Wang et al. developed a passive upper-limb exoskeleton with a simplified design to reduce the muscle activity required for repetitive lifting tasks, thereby reducing the risk of sprains and strains (117). Additionally, the design of ergonomically optimized hand tools has the potential to mitigate unintentional injuries such as contusions, scratches, sprains, and strains while simultaneously enhancing worker productivity. Hence, it is crucial for optimal design properties to prioritize physical body characteristics, flexibility of removal, simplification, and safety during usage. Moreover, integrating new education intervention technology would enhance motivation toward adopting safe practices in education. For example, Namkoong et al. developed an AR 3D simulator called Augmented Reality Intervention for Safety Education (ARISE) in order to promote the level of farm agricultural safety education (118). ARISE technology presents farm accident situations and provides second-hand experience of incidents that frequently occur on farms using immersive media technology for farmers and their families.

When the event occurs, the implementation of more effective protective measures, such as improved respiratory protection, an elastomeric honeycomb helmet, a modified motorized sprayer, and a vehicle’s protective structure, could mitigate fatal injuries. Considering the variations in the individual body dimensions, the design of PPE incorporates anthropometric data that are specific to agricultural workers. This innovative ergonomic design of PPE is illustrated to increase safety and comfort while enhancing operators’ efficiency (119). Simultaneously, research and development efforts focused on newer machinery equipped with built-in safety features have proven effective in reducing injury severity among farmers (120). The adoption of innovative models such as tractors featuring an ROPS and power take-off (PTO) guards has been associated with a significant decrease in fatal injuries (121). For example, Bruno et al. utilized a kinematic model to predict vehicle orientation and stability on idealized slopes (77). Innovative designs for agricultural vehicles can optimize their operation on a sloping ground and minimize the risk of collision. Moreover, it is crucial to enhance the design and improvement of escape routes and clear farm roads in order to mitigate more severe injuries. However, there is some evidence suggesting that farmers may inhibit the use of this innovative equipment due to economic constraints, lack of knowledge, and behavioral resistance to changing habits by the farming community, especially on small-area farms (122, 123).

#### 3.4.3 Enforcement

The implementation of stringent and effectively enforced universal legislation, regulations, and policies about farming has proven its efficacy as a comprehensive strategy in reducing the overall burden of farm-related injuries (124). However, the health and rights of small-scale farmers and self-employed family farming remain uncertain due to their exclusion from government policies and support (125). Before the event, imperfections in legislation concerning usage of firearms, hazardous material handling, and storage practices, as well as pesticide management have persistently posed challenges. Enforcing laws related to firearms is instrumental in safeguarding youth against unintentional or self-inflicted firearm-related injuries and fatalities (37). Moreover, promoting responsible firearm ownership explicitly provides robust protection for families or other relevant adults against accidental firearm discharge or farmer suicide (126). Moreover, implementing restrictions on the purchase amount of several medicines such as paracetamol and aspirin in a single transaction, alongside prominently displaying overdose on the packaging, has been proven effective in preventing instances of poisoning and suicides (52).

Furthermore, there is a pressing need to improve policies and legislation aimed at safeguarding farmers, such as mandatory usage of helmets and PPE and stricter regulations on drink and driving. Implementing these enforcement measures could effectively enhance agricultural safety and reduce the occurrence of accidents. For instance, in 1962, primary legislation was enacted to make motorcycle helmets and seatbelts compulsory in England, and this law was later adopted in 1973. Motorcyclists who failed to wear helmets would pay penalty charges with non-payment potentially leading to imprisonment (53). Additionally, Australia introduced legislation in 1985, requiring ROPS to be installed on new tractors. The introduction of this legislation was associated with a decrease in fatal injuries (54).

Even if farmers suffer agricultural injuries, they often resume work before achieving full recovery, thereby potentially leading to a cycle of damage (55). In fact, treatment and definitive management process of agricultural injuries suffer from time delays, which can be detrimental to farmers’ health. Therefore, admissible management policies should be provided by the public sector for injured farmers, such as implementing a work rotation system, providing appropriate breaks during working hours, conducting routine annual medical examinations of workers, and ensuring access to health services (127). Additionally, adopting administrative measures to enhance farm safety and minimize workplace hazards should include regular mandatory and voluntary risk assessments, routine and unannounced inspections, regulation of construction requirements, and machinery conditions (128). It is worth noting that these management practices and legislation should be progressively improved through concerted multisectoral efforts.

#### 3.4.4 Economic incentives

The government can utilize various financial measures to incentivize farmers to prevent injuries, such as subsidizing occupational safety compensation insurance for farmers, allocating funds for the improvement of working conditions, covering healthcare service expenses, and mandating educational programs. In the pre-event stage, subsidizing investments in occupational safety education and training would also be effective. For example, European public authorities have enhanced safety standards on farms by increasing both financial and non-financial resources allocated toward safety production, training and education courses, and stakeholder engagements (129).

In the event stage, by investing in technology, safety equipment, and construction, the physical environment of the workplace can be optimized to effectively reduce the risks of injury. A study showed that farms that receive sufficient investment in infrastructure, machinery, and equipment reduced injury risks for farmers (130). For example, providing music or radio programs and designated rest areas can alleviate fatigue among workers.

In post-event stage, medical expenses and loss of income due to injuries can lead to impoverishment. For small-scale farmers who cannot afford insurance premium for occupational injury, private or commercial health insurance subsidized by the government or union is considered a protective factor after an injury compared with individually purchased policies (131). The South Korean Government’s policy of providing more than half of subsidy for farmers’ occupational safety insurance has contributed to protecting their health and maintaining safe work environments (132).

#### 3.4.5 Emergency response

In the pre-event stage, ensuring reliable surveillance is critical for preventing farmers’ injuries. For example, it is imperative to establish and refine national and community surveillance systems that encompass natural hazards, toxic chemicals, and agriculture-related injuries in order to predict hazardous situations in farming activities (133). Currently, researchers are designing electronic surveillance systems to track farm injury that utilizes several match-merging data sources (134). Moreover, there is an urgent need for an injury monitoring mechanism tailored for farmers to identify and report poisonings, falls, and agricultural vehicle collisions. Implementing agricultural injury surveillance systems can facilitate the collection of pertinent information and promote epidemiologic studies to find exposure scenarios and risk factors (135). Simultaneously, the researchers emphasize the urgent need to improve the quality, timeliness, and flexibility of surveillance to effectively prevent high-risk situations (136). For farmers, effective task scheduling, regular inspection of tool and machinery safety, meticulous planning, and preparation for daily farming activities are crucial aspects of task management and personal safety maintenance (66). Furthermore, adequate preparation and training for rural healthcare workers and medical resources are also imperative. Generally speaking, the harvest season is associated with an increased rate of injuries due to seasonal variations in agricultural tasks (137). Therefore, rural healthcare providers should ensure sufficient preparedness during periods of high injury incidence. However, in America, rural areas face a shortage of healthcare professionals, hospitals, and intensive care units (ICUs). Rural health services such as emergency medical services (EMSs), community pharmacists, and home health agencies exhibit lower levels of readiness compared with urban areas (138).

In the event stage, the emergency response capability covering the primary agricultural workforce can be manifested by the government effort and medical service. Through government and media offering consulting services, farmers can easily comprehend risks and, subsequently, take more efficient actions before and after injuries occur. In addition, it is crucial to establish accessible communication channels for effective risk communication, such as television, radio, social media platforms, videos, and interpersonal communication. It should be noted that rural and remote farms often receive less timely risk communication compared with other areas, as evidenced by studies conducted in Australia (139).

In the post-event stage, the management of patients depends on the accessibility of public health and medical service facilities. For example, geographical remoteness between healthcare services and farms and limited clinical service capacity at rural hospitals pose disadvantages to farmers’ risk management (140). Emergency medical services such as Helicopter Emergency Medical Service (HEMS), Ground Emergency Medical Service (GEMS), and telehealth play a crucial role in emergency care. Particularly in rural or remote farming areas with limited resources, HEMS can significantly reduce rescue time, improve patient outcomes, and decrease mortality following severe trauma (92, 93, 141). In addition, telemedicine supports various conditions and services, including health promotion, primary care, medical and surgical interventions, and rehabilitation through video or telephone consultations (94). Its benefits for patients included ease of scheduling and reduced wait time for appointments, quicker responses to emergencies, and decreased travel time and costs. Efficient risk assessment and task management are also important for safe decision-making (110). For instance, conducting longitudinal risk assessment on individual risk factors, hazard materials, and farm conditions can aid in preventing injuries. Thus, the establishment of self-assessment programs in farm safety could facilitate behavioral changes among farmers and encourage the adoption of safer farming practices, ultimately leading to a reduction in the number of farm accidents and injuries.

## 4 Discussion

In this study, there was a significant increase in the number of studies, indicating an increased research focus on farmer injuries. Moreover, the United States has made substantial efforts in this field, which can be attributed to its robust injury surveillance system and comprehensive public health service (95). Co-occurrence analysis of keywords revealed a higher frequency of age-related terms such as “middle aged,” “young adult,” “80 and older,” and “age factors.” Age factors have consistently been a focal point for researchers. Numerous studies have indicated that young adults (13) and middle-aged farmers face a high risk of serious injury (113). Young adult farmers are generally more susceptible to non-fatal injuries due to their inclination toward risk-taking behaviors and a greater propensity for seeking thrilling experiences. On the other hand, middle-aged farmers tend to engage in high-risk activities such as heavy workloads, managing employee relations, coping with family pressures, and facing challenges related to unaffordable healthcare. However, older farmers experience higher fatality rates and severity of injuries. Consequently, without meaningful and effective interventions specifically tailored for different age groups of farmers, both the economic and human costs associated with these injuries are likely to escalate. Additionally, the term “environmental and occupational health” also has a high frequency. In recent years, an increasing number of studies have identified the significance of ensuring workplace safety by considering factors such as workplace design, off-farm work hours, and outdoor conditions (10). The farmers play a crucial role in relation to regulations and fostering a safe workplace climate when discussing the risks of injuries (96).

Despite efforts made to eliminate many risk factors associated with farm-related injuries at their source, eliminating these hazards is challenging due to the complex interaction of multiple risk factors. In this study, we primarily utilized the Haddon matrix, which takes into account four aspects (host, agent, physical environment, and social environment) and three phases (pre-event, event, and post-event), to identify the risk factors that contribute to farmer injuries. Additionally, we investigated strategies for reducing these injuries. The Haddon matrix not only guides epidemiological research but also aids in developing effective interventions. At an individual level, it is worth noting that there is an increasing trend in the average age of farmers who continue working beyond normal retirement age (142). In general, older farmers are confronted with changes in their physical characteristics (e.g., loss of balance, reduced strength and agility, and slower reaction times). They also experience decreased tolerance to fatigue and regular medication use, all of which contribute to an increased risk profile for fatal injuries. Moreover, due to a lack of education regarding risk awareness, they are less inclined to practice tillage or operate machinery using proper protective devices following correct guidelines. In fact, farmers often suffer from sleep deprivation as they need to rise early for work (84). Under conditions of inadequate sleep and high levels of stress, farmers may experience reduced attention and concentration. This can lead to the adoption of unsafe behaviors that easily result in errors and accidents during machine operation over extended periods, handling large animals, or working at elevated heights (143). The consumption of alcohol or certain medications such as tranquilizers, sleeping pills, and antidepressants may induce behavioral changes and impair farmers’ alertness and judgment while performing complex tasks. As a result, this increases the risks of crash-related or fall-related injuries (61). However, in order to facilitate behavioral changes among farmers, it is imperative to enhance their perception and willingness. A survey conducted among farmers in South Korea revealed that over half of them expressed a desire for agricultural health and safety education; however, they have had limited access to such educational opportunities (41). Consequently, it is crucial for the government and local communities to actively promote comprehensive safety education for farmers through various channels including village schools, radio broadcasts, and social media platforms.

At the host level, numerous studies have been published on pesticide poisoning among farmers. For instance, various pesticides (such as herbicides, CPF, profenofos, ethion, and malathion) and other toxic agrochemical substances can directly impact neural systems (144). The global use of toxic agrochemicals such as pesticides in agriculture has increased due to their cost-effectiveness, high efficiency, and easy accessibility (145). Evidence indicates that factors influence pesticide exposure levels such as duration of farm work, mixing multiple highly toxic pesticides, personal spraying habits and hygiene practices at work sites, and inadequate use of PPE (146–148). The management of toxic substance storage and regular safety education should be institutionalized through various means, such as leveraging social networks and farmer field schools. Furthermore, the utilization of safe farm motor vehicles, in conjunction with appropriate protective equipment for both individuals and vehicles, plays a crucial role in mitigating injuries. A study demonstrated that farmers who did not utilize any PPE had 5.7 times higher risk of injury compared with those who used at least one piece of PPE (149). In practice, the inadequate or improper use of PPE by farmers is a prevalent occurrence in agricultural settings. For instance, a review highlighted that compliance rates for wearing helmets and seatbelts among farmers range from 30 to 70% (150). Therefore, it is crucial to prioritize education on appropriate PPE usage, alongside implementing legal and systematic management approaches, in order to develop effective strategies for preventing injuries. Additionally, there is a need to explore and develop innovative safety designs for protecting equipment such as elastomeric honeycomb helmets and ROPS on tractors, to mitigate injuries, resulting from farm vehicle collisions. Furthermore, the limited medical resources and weaker health systems in rural areas pose significant challenges to injury recovery (151). Hence, it is crucial for governments to allocate financial resources toward enhancing the development of healthcare infrastructure in rural regions.

At the environmental level, inadequate physical workplace conditions and farm road infrastructure may contribute to an increase in safety hazards and unintentional injuries such as falls and collisions involving farm vehicles. Numerous studies have highlighted that factors such as poor road lighting, narrowness, and ruggedness of farm roads are associated with a higher risk of accidents (35). Furthermore, there is a gradually significant interaction effect between individual physiological factors and agricultural work settings/equipment. Research investigating the relationship between these factors could greatly benefit efforts to improve safety. Therefore, it is crucial for professionals to provide occupational safety and health services or consultation to agricultural workers in order to improve their working conditions and meet relevant standards. Most of all, considerations should be given to both farmers’ acceptance and positive cost-to-benefit ratio when devising effective safety interventions (140). Meanwhile, it is crucial to prioritize the establishment of evacuation pathways and safe havens in order to mitigate avoidable injuries. Government and relevant departments should develop comprehensive guidance documents and provide targeted financial support for the construction of safe workplaces. After an injury occurs, it is imperative that injured farmers have rapid access to trauma services. Over the past two decades, there has been a substantial increase in the utilization of Helicopter Emergency Medical Services (HEMSs) for transporting severely injured trauma patients. Helicopters can be particularly advantageous in remote farms that are not easily accessible by ground ambulances (152). They enable experienced medical teams to promptly reach complex patients and offer advanced resources. However, it should be noted that HEMS operations tend to incur higher costs compared with Ground Emergency Medical Services (GEMSs) (153). Hence, GMES and telehealth play an indispensable and pivotal role in the provision of medical services for non-fatal injuries. Furthermore, within a familial agricultural context, young workers’ engagement in farming tasks is influenced by their families, potentially leading to the adoption of unsafe risk-taking behaviors during adulthood. Therefore, targeted guidance programs should also emphasize strengthening parental and peer support. Overall, it is essential to prioritize safety measures that encompass ergonomic engineering controls, comprehensive safety education and training programs, administrative protocols, and innovative technology advancements.

However, the current studies were limited to the screening of influencing factors, with few studies exploring the interrelationship between risk factors. Furthermore, analysis of long-term consequences after injuries for farmers is still deficient. Additionally, the studies about economic incentives, especially from government and emergency response system targeted to farm-related injuries, were inadequate. Therefore, future research should focus on investigating effective injury formation mechanisms to enhance farm safety. Moreover, collaborative efforts among multi-institution are imperative for developing appropriate injury prevention strategies.

## 5 Conclusion

In summary, there has been a noticeable upward trend in the number of literature related to the risk factors of farmer injuries from 2003 to 2023. The current research focus revolves around age-related factors and occupational and environmental factors. The identification of key risk factors for farmer injuries encompasses four aspects (host agent, physical environment, and social environment) across three phases (before-event, event, and post-event). Corresponding interventions have been summarized as effective measures for preventing, reducing, or mitigating injuries. These measures include education programs, innovative engineering designs, government regulations enforcement along with legal frameworks, economic incentives, and improved emergency response strategies. Our study provides a comprehensive foundation for the investigation and prevention of injuries among farmers. However, tailored interventions should be implemented based on specific local conditions.

## Author contributions

XuQ: Writing – original draft, Conceptualization, Data curation, Software, Writing – review & editing. XuY: Data curation, Investigation, Writing – original draft. XC: Software, Writing – review & editing. SL: Software, Writing – review & editing. MH: Data curation, Writing – review & editing. ZT: Investigation, Writing – review & editing. XiY: Validation, Writing – review & editing. XiQ: Supervision, Writing – review & editing. FS: Conceptualization, Supervision, Writing – review & editing. SW: Conceptualization, Project administration, Writing – review & editing.

## Footnotes

^1^https://data.worldbank.org.cn/
