# Supplementary material for: Profile and risk factors in farmer injuries: a review based on Haddon matrix and 5 E’s risk reduction strategy
Source: Front Public Health. 2024 Jun 6;12:1322884. doi: 10.3389/fpubh.2024.1322884 (PMC11187248; doi:10.3389/fpubh.2024.1322884)
Supplement: Supplementary file 1 [file Table_1.DOCX]

| Countries | Numbers |
| --- | --- |
| The United States | 201 |
| Australia | 96 |
| Canada | 63 |
| Italy | 45 |
| Germany | 43 |
| Finland | 31 |
| United Kingdom | 28 |
| Ireland | 27 |
| Korea | 23 |
| New Zealand | 19 |
| France | 16 |
| Switzerland | 15 |
| Netherlands | 14 |
| Japan | 12 |
| Poland | 10 |
| Denmark | 10 |
| Norway | 8 |
| Belgium | 6 |
| Singapore | 6 |
| Austria | 5 |

Table S The top 20 countries by number of publications
